# Selective inhibition of protein kinase C *β*_2_ attenuates the adaptor P66^Shc^-mediated intestinal ischemia–reperfusion injury

**DOI:** 10.1038/cddis.2014.131

**Published:** 2014-04-10

**Authors:** Z Chen, G Wang, X Zhai, Y Hu, D Gao, L Ma, J Yao, X Tian

**Affiliations:** 1 Department of General Surgery, Second Affiliated Hospital, Dalian Medical University, 116023 Dalian, China; 2 Department of Pharmacology, Dalian Medical University, 116044 Dalian, China

**Keywords:** PKC*β*_2_, p66^Shc^, oxidative stress, apoptosis, intestinal ischemia reperfusion

## Abstract

Apoptosis is a major mode of cell death occurring during ischemia–reperfusion (I/R) induced injury. The p66^Shc^ adaptor protein, which is mediated by PKC*β*, has an essential role in apoptosis under oxidative stress. This study aimed to investigate the role of PKC*β*_2_/p66^Shc^ pathway in intestinal I/R injury. *In vivo*, ischemia was induced by superior mesenteric artery occlusion in mice. Ruboxistaurin (PKC*β* inhibitor) or normal saline was administered before ischemia. Then blood and gut tissues were collected after reperfusion for various measurements. *In vitro*, Caco-2 cells were challenged with hypoxia–reoxygenation (H/R) to simulate intestinal I/R. Translocation and activation of PKC*β*_2_ were markedly induced in the I/R intestine. Ruboxistaurin significantly attenuated gut damage and decreased the serum levels of tumor necrosis factor-*α* (TNF-*α*) and interleukin-6 (IL-6). Pharmacological blockade of PKC*β*_2_ suppressed p66^Shc^ overexpression and phosphorylation in the I/R intestine. Gene knockdown of PKC*β*_2_ via small interfering RNA (siRNA) inhibited H/R-induced p66^Shc^ overexpression and phosphorylation in Caco-2 cells. Phorbol 12-myristate 13-acetate (PMA), which stimulates PKCs, induced p66^Shc^ phosphorylation and this was inhibited by ruboxistaurin and PKC*β*_2_ siRNA. Ruboxistaurin attenuated gut oxidative stress after I/R by suppressing the decreased expression of manganese superoxide dismutase (MnSOD), the exhaustion of the glutathione (GSH) system, and the overproduction of malondialdehyde (MDA). As a consequence, ruboxistaurin inhibited intestinal mucosa apoptosis after I/R. Therefore, PKC*β*_2_ inhibition protects mice from gut I/R injury by suppressing the adaptor p66^Shc^-mediated oxidative stress and subsequent apoptosis. This may represent a novel therapeutic approach for the prevention of intestinal I/R injury.

Critical massive intestinal ischemia occurs in response to conditions such as acute mesenteric thrombotic or embolic occlusion, which are associated with high mortality.^1, 2^ Other gut ischemia cases followed by hemorrhagic shock, volvulus, sepsis, and abdominal aortic aneurysm surgery have a more subtle but no less damaging injury. Although restoration of the blood supply to the ischemic gut is critical to salvage, the reperfusion may paradoxically aggravate ischemic tissue damage and systemic inflammatory response.^3^ During the reperfusion period, a vicious cascade occurs including massive reactive oxygen species (ROS) generation, the activation of pro-apoptotic factors, and systemic inflammatory responses such as cytokine/chemokine release and polymorphonuclear neutrophil infiltration.^4, 5^ It becomes recognized that oxidative stress-induced ischemia/reperfusion (I/R) damage involves multiple signaling pathways.

PKC, a family of serine/threonine protein kinases comprising at least 12 members, has a central role in signal transduction and intracellular crosstalk.^6^ PKC*β*_1_ and PKC*β*_2_ isoforms are encoded by the same gene, PKC*β*, and are not expressed in homozygous PKC*β*^−/−^ mice (NCBI Gene Database, identification number 18751). Multiple PKC isozymes are expressed in the intestine.^7^ Gene deletion or pharmacological blockade of PKC*β* protects ischemic myocardium, decreases infarct size, and enhances recovery of ventricular function.^8^ Homozygous PKC*β*-null mice and WT mice fed with ruboxistaurin (LY333531, selective PKC*β* inhibitor) and subjected to single-lung I/R display increased survival, indicating that PKC*β* has a pivotal role in the I/R-induced apoptosis.^9^ Despite these observations, the underlying mechanism by which PKC*β* exerts deleterious effects in the intestinal I/R remains unclear.

The Shc adaptor protein family, consisting of the p66^Shc^, p52^Shc^, and p46^Shc^ isoforms, is encoded by the ShcA locus.^10^ Due to the presence of a unique N-terminal domain (CH2), which is required for redox activity, p66^Shc^ is the only isoform that acts as a redox enzyme implicated in mitochondrial ROS generation and the translation of oxidative signals into apoptosis.^11^ Phosphorylation at Ser36 of p66^Shc^ is required for conferring increased susceptibility to oxidative stress, and is critical for the cell apoptosis elicited by oxidative damage.^12^ Migliaccio *et al.*^11^ reported the p66^Shc−/−^ mouse increased resistance to oxidative stress and extended lifespan by 30%. Deletion of the p66^Shc^ gene in mice is shown to protect hind limb,^13^ brain,^14^ and *ex vivo* hearts^15^ from I/R injury. It suggests that p66^Shc^ would be a target to decrease the injury caused by intestinal I/R.

Hydrogen peroxide (H_2_O_2_) and hyperglycemic stress activate the PKC*β*_2_ isoform to induce p66^Shc^ phosphorylation at Ser-36, allowing transfer of the adaptor protein from the cytosol to the inner mitochondrial membrane, where it amplifies oxidative stress and catalyzes ROS production via cytochrome c oxidation.^16, 17, 18^ Therefore, we hypothesize that there may be a PKC*β*_2_/p66^Shc^ signaling pathway in the pathogenesis of intestinal I/R.

## Results

### Membrane translocation and phosphorylation of PKC*β*_2_ in response to intestinal I/R

**Figure 1:**
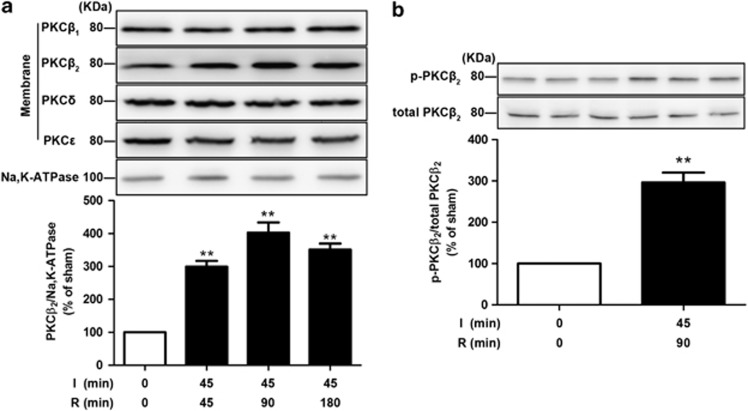
Intestinal I/R-mediated membrane translocation and phosphorylation of PKC*β*_2_. Mice were subjected to 45 min ischemia followed by 45, 90, or 180 min reperfusion. (**a**) Representative western blot demonstrating the expression of PKC*β*_1_, PKC*β*_2_, PKC*δ*, and PKC*ɛ* in membranous fractions with Na,K-ATPase as a loading control. (**b**) Representative western blot demonstrating p-PKC*β*2 (Thr 641) and total-PKC*β*2 expression from sham and 90 min reperfusion intestine. All results are expressed as means±S.E.M., *n*=3 per group, **P<0.01 *versus* sham

To test the hypothesis that PKC could be activated by I/R injury, we assessed cell membranous fraction of patterns for distinct PKC isoforms in the intestinal tissue subjected to 45 min ischemia followed by 45, 90, or 180 min reperfusion. A selective membrane translocation of PKC*β*_2_ was detected, whereas PKC*β*_1_, PKC*δ*, and PKC*ɛ* showed no differences in membrane fraction after various reperfusion times (Figure 1a), indicating that PKC*β*_2_ is specifically activated by I/R. To support this notion, we detected that a 90-min reperfusion significantly increased PKC*β*_2_ phosphorylation at the thr-641 residue, leading to a markedly increased ratio of phosphorylated PKC*β*_2_/total PKC*β*_2_ (Figure 1b). These results demonstrated that both membrane translocation and activation of PKC*β*_2_ occurred in the model of intestinal I/R.

### Ruboxistaurin attenuates gut damage and the systemic inflammatory response after intestinal I/R

**Figure 2:**
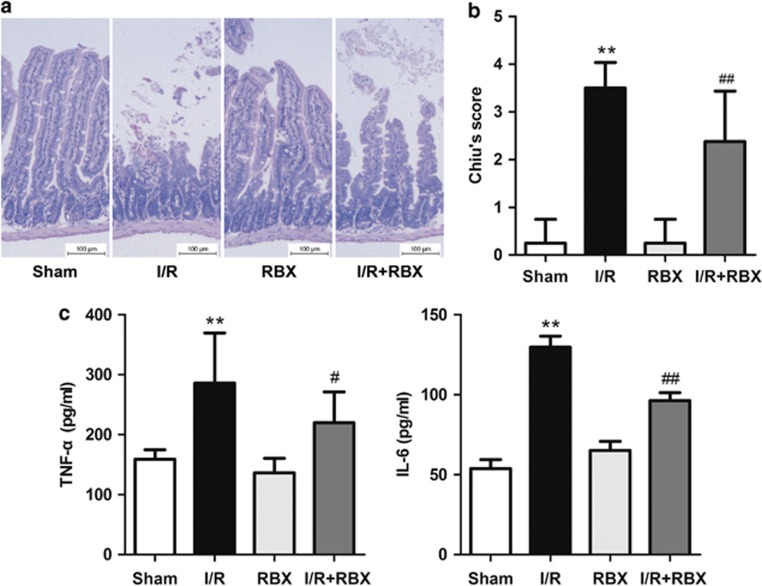
Ruboxistaurin pretreatment decreases the gut injury and the systemic inflammatory response after intestinal I/R. Normal saline and ruboxistaurin were given before sham and 45 min ischemia followed by 90 min reperfusion. (**a**) Gut tissues harvested after intestinal I/R were stained with hematoxylin and eosin, and examined under light microscopy at × 400 magnification. Representative images for sham, I/R, sham ruboxistaurin pretreatment, and I/R ruboxistaurin pretreatment groups. (**b**) Histologic injury scores of the gut in different groups were quantified as described in Materials and Methods. (**c**) Serum levels of TNF-*α* and IL-6 were determined by ELISA after intestinal I/R. All results are expressed as means±S.E.M., *n*=8 per group, **P<0.01 *versus* sham; ^##^*P<*0.01, ^#^*P<*0.05 *versus* I/R. RBX, ruboxistaurin

Next, ruboxistaurin (oral PKC*β* inhibitor) and normal saline were given as a pretreatment before the superior mesenteric artery was occluded for 45 min followed by 90 min reperfusion. On examination of the histological changes, ruboxistaurin preserved the integrity of morphological structure well, and reduced both hemorrhage and neutrophil infiltration in the I/R intestine (Figure 2a). Similarly, the gut histological injury scores were significantly increased following I/R injury *versus* sham, and was reduced by ruboxistaurin (Figure 2b). Additionally, intestinal I/R significantly increased the serum levels of tumor necrosis factor-*α* (TNF-*α*) and interleukin 6 (IL-6). Ruboxistaurin, however, almost abrogated the increase in TNF-*α* and IL-6 concentrations (Figure 2c).

### Ruboxistaurin suppresses intestinal I/R-induced activation of PKC*β*_2_ and p66^Shc^

**Figure 3:**
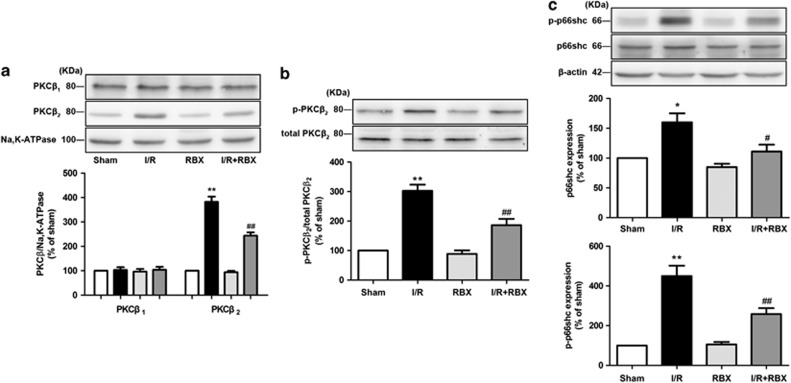
The effects of ruboxistaurin (oral PKC*β* inhibitor) upon membrane distributions of PKC*β*_1_ and PKC*β*_2_, expression levels of p-PKC*β*_2_ (Thr 641) and total-PKC*β*_2_, and expression levels of p-p66^Shc^ (Ser 36) and total-p66^Shc^ in intestinal tissue. (**a**) Representative western blot demonstrating PKC*β*_1_ and PKC*β*_2_ expression in membrane fractions with Na,K-ATPase as a loading control. (**b**) Representative western blot demonstrating p-PKC*β*_2_ and total-PKC*β*_2_ expression. (**c**) Representative western blot demonstrating p-p66^Shc^ and total-p66^Shc^ expression with *β*-actin as a loading control. All results are expressed as means±S.E.M., *n*=3 per group, **P<0.01, *P<0.05 *versus* sham; ^##^*P<*0.01, ^#^*P<*0.05 *versus* I/R. RBX, ruboxistaurin

Figure 3a showed that ruboxistaurin greatly suppressed the translocation of PKC*β*_2_ in the I/R intestine over the same time period in which PKC*β*_1_ was not impacted. Meanwhile, ruboxistaurin prevented the intestinal I/R-induced increase in the phosphorylation of PKC*β*_2_ without affecting the expression of total PKC*β*_2,_ and suppressed the increased ratio of phosphorylated PKC*β*_2_/total PKC*β*_2_ (Figure 3b). Intestinal I/R moderately increased the expression of p66^Shc^, and greatly induced p66^Shc^ phosphorylation. However, ruboxistaurin significantly reduced I/R-induced p66^Shc^ overexpression and phosphorylation at ser36 (Figure 3c). Therefore, our study indicated that ruboxistaurin inhibited both PKC*β*_2_ activation and PKC*β*_2_-mediated p66^Shc^ activation in the I/R intestine.

### Hypoxia/reoxygenation or phorbol 12-myristate 13-acetate-induced p66^Shc^ activation: involvement of PKC*β*_2_

**Figure 4:**
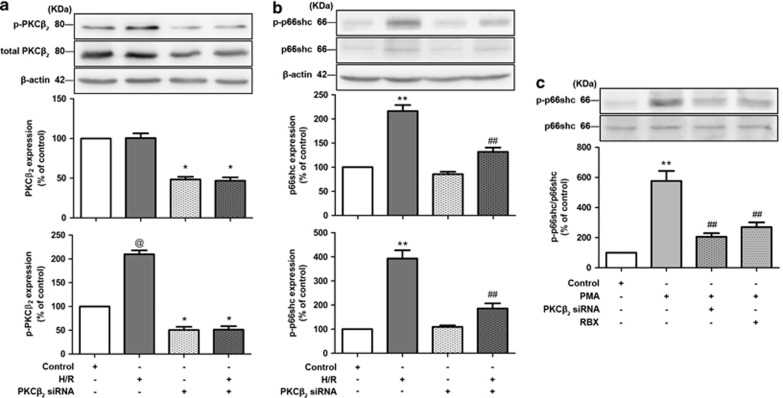
Expression of PKC*β*_2_ and p66^Shc^ in cultured Caco-2 cells following various treatments under normoxic or H/R conditions and PMA exposure. Caco-2 cells were incubated under hypoxic conditions for 15 h and then cultured under normoxic conditions for 6 h reoxygenation. Scrambled siRNA was used as a negative control. Representative western blot demonstrating (**a**) p-PKC*β*2 and total-PKC*β*_2_, and (**b**) p-p66^Shc^ and total-p66^Shc^ expression with *β*-actin as a loading control in Caco-2 cells transfected with PKC*β*_2_ siRNA, exposed to normoxic or H/R conditions. (**c**) Representative western blot demonstrating p-p66^Shc^ and total-p66^Shc^ expression with *β*-actin as a loading control in Caco-2 cells transfected with PKC*β*_2_ siRNA, exposed to PMA. All results are expressed as means±S.E.M., *n*=3 per group, **P<0.01, *P<0.05 *versus* sham; ^##^*P<*0.01 *versus* I/R; ^@^*P*<0.05 *versus* all other groups

Hypoxia/reoxygenation (H/R) of cells *in vitro* is a simple model of organ I/R, at least partly reflecting the pathophysiology *in vivo*. To simulate *in vivo* intestinal I/R, Caco-2 cells were exposed to H/R. To determine whether PKC*β*_2_ is specifically required for the activation of p66^Shc^, we suppressed its expression using human-specific PKC*β*_2_ small interfering RNA (siRNA) under normoxic and H/R conditions. Knockdown of PKC*β*_2_ by siRNA reduced the expression of PKC*β*_2_ and its phosphorylation in Caco-2 cells under normoxic and H/R conditions (Figure 4a). Our data showed that PKC*β*_2_-siRNA had no effects upon p66^Shc^ activation under normoxic conditions, but prevented p66^Shc^ overexpression and phosphorylation under H/R conditions (Figure 4b). To further confirm whether p66^Shc^ activation was activated by PKC*β*_2_, we examined the effect of phorbol 12-myristate 13-acetate (PMA), a classical PKC activator, on the activation of p66^Shc^. The exposure of PMA markedly increased p66^Shc^ phosphorylation in Caco-2 cells, which was inhibited significantly by PKC*β*_2_ siRNA and ruboxistaurin (Figure 4c).

### Inhibition of PKC*β*_2_ activation by ruboxistaurin attenuates gut oxidative stress after intestinal I/R

**Figure 5:**
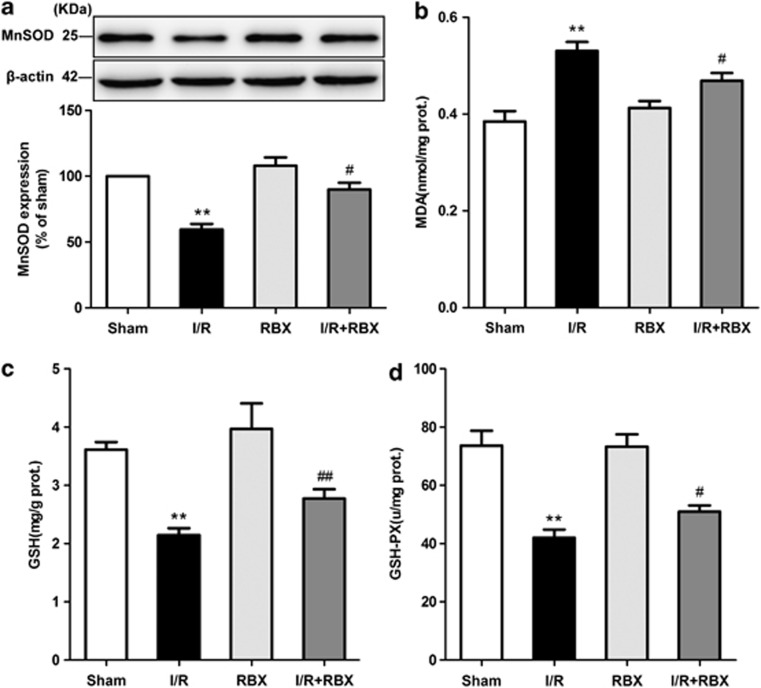
Inhibition of PKC*β*_2_ activation by ruboxistaurin attenuates gut oxidative stress after intestinal I/R. (**a**) Representative western blot demonstrating MnSOD protein expression (*n*=3). (**b**) The activity of MDA in the intestine was determined. (**c**) The GSH levels in the intestine. (**d**) The GSH-PX levels in the intestine (*n*=8 per group for **b**, **c**, and **d**). All results are expressed as means±S.E.M., **P<0.01 *versus* sham; ^##^*P<*0.01, ^#^*P<*0.05 *versus* I/R

To evaluate the oxidative state of the gut after I/R, we measured the levels of manganese superoxide dismutase (MnSOD), glutathione (GSH), glutathione peroxidase (GSH-PX), and malondialdehyde (MDA) in the intestinal tissues. Ruboxistaurin reversed intestinal I/R-induced anti-oxidant enzyme MnSOD downregulation (Figure 5a). ROS accumulation was increased in intestinal I/R tissue based on the assessment of MDA activity, which was reduced by ruboxistaurin (Figure 5b). In parallel, ruboxistaurin preserved intestinal I/R-induced GSH exhaustion and GSH-PX activity reduction (Figures 5c and d). Taken together, these data indicated that blockade of PKC*β*_2_ decreased gut oxidative stress after intestinal I/R.

### Inhibition of PKC*β*_2_ activation by ruboxistaurin inhibits gut apoptosis after intestinal I/R

**Figure 6:**
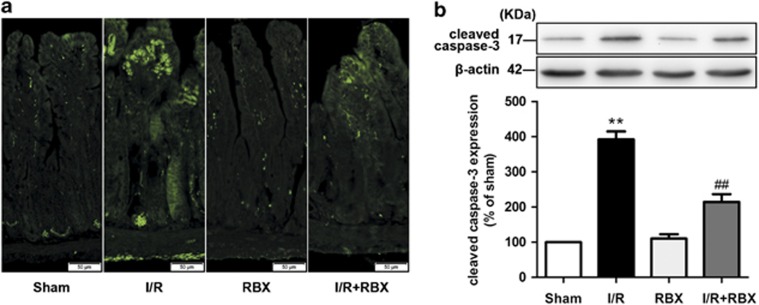
Inhibition of PKC*β*_2_ activation by ruboxistaurin inhibits gut apoptosis after intestinal I/R. (**a**) TUNEL staining of paraffin-embedded intestinal tissue sections. Representative images for sham, I/R, sham ruboxistaurin pretreatment, and I/R ruboxistaurin pretreatment groups (*n*=8). (**b**) Representative western blot demonstrating cleaved caspase-3 protein expression. All results are expressed as means±S.E.M., *n*=3 per group, **P<0.01 *versus* sham; ^##^*P<*0.01 *versus* I/R

To determine the apoptosis state of the gut after I/R, a terminal deoxynucleotidyl transferase mediated deoxyuridinetriphosphate nick end labeling (TUNEL) assay was conducted. The apoptotic cells in the gut were elevated from non-detectable to well observed after intestinal I/R, whereas ruboxistaurin significantly reduced the number of apoptotic cells (Figure 6a). In addition, ruboxistaurin significantly suppressed the increased levels of cleaved caspase-3, another marker of cell apoptosis, in the I/R intestinal tissue (Figure 6b).

## Discussion

In the present study, we have demonstrated that I/R-induced intestinal dysfunction involved the PKC*β*_2_/p66^Shc^ signaling pathway. PKC*β*_2_ activation played an essential role in the pathogenesis of intestinal I/R injury, and inhibition of excessive activation of PKC*β*_2_ by ruboxistaurin reduced intestinal I/R injury at least partly via attenuation of the p66^Shc^ activation. P66^Shc^ acted as a redox enzyme implicated in mitochondrial ROS generation and the translation of oxidative signals into apoptosis. We provided evidence that pharmacological blockade or gene knockdown of PKC*β*_2_ inhibited I/R-induced p66^Shc^ activation, demonstrating that excessive p66^Shc^ activation is associated with PKC*β*_2_ activation. To the best of our knowledge, this is the first study examining the relationship between PKC*β*_2_ and p66^Shc^ in intestinal I/R.

Previous studies have reported activation of PKC*β*_2_, PKC*δ*, and PKC*ɛ* in cardiac ischemia or I/R,^8, 19, 20^ activation of PKC*β*_2_ associated with the response to single-lung I/R,^9^ and activation of PKC*δ* and PKC*ɛ* related to cerebral I/R.^21^ Our results demonstrated that the activated principal isoform of PKC in intestinal I/R was specifically PKC*β*_2_, not PKC*β*_1_, PKC*δ*, or PKC*ɛ* (Figures 1a and b). These data suggested that the activation of individual PKC isoforms in ischemia or I/R is tissue specific. Moreover, our results indicated that in intestinal I/R, ruboxistaurin did not change the translocation of PKC*β*_1_ over the same time period in which PKC*β*_2_ was greatly impacted (Figure 3a). Taken together, it is likely that the primary role of ruboxistaurin was to inhibit the activation of PKC*β*_2_ in intestinal I/R.

H/R significantly induces the activation of p66^Shc^, and ablation of p66^Shc^ is cytoprotective against oxidative stress and apoptosis in hepatocytes.^22^ This may be clinically relevant as the mRNA level of p66^Shc^ is increased in peripheral blood mononuclear cells of patients with acute myocardial infarction.^23^ In human aortic endothelial cells, selective inhibitor of PKC*β*_2_ prevented p66^Shc^ activation after exposed to hyperglycemic stress or oxidized low-density lipoprotein, respectively.^18, 24^ Our data showed that inhibition of PKC*β*_2_ activation by ruboxistaurin attenuated p66^Shc^ overexpression and phosphorylation at ser36 in the I/R intestine (Figure 3c). *In vitro* studies, knocking down PKC*β*_2_ via siRNA inhibited the activation of PKC*β*_2_, and further prevented p66^Shc^ overexpression and phosphorylation under H/R conditions (Figure 4). By using both pharmacological blockade and gene knockdown PKC*β*_2_ *in vivo* and *in vitro* experiments, we tested the above hypothesis that there may be a PKC*β*_2_/p66^Shc^ signaling pathway in intestinal I/R.

Gut I/R produces excessive amounts of ROS, which is responsible for the intestinal mucosa damage.^25^ Given exposure to ROS, mitochondrial proteins, lipids, and DNA are believed to be primary targets of oxidative damage, leading to alteration or loss of cellular functions, and causing inhibition of proliferation and induction of apoptosis.^26^ A growing body of evidence links p66^Shc^ to oxidative stress as the adaptor protein has a pivotal role in modulating the intracellular redox state, increasing susceptibility to oxidative stress, and resulting in apoptosis elicited by oxidative damage.^27, 28, 29^ Our data demonstrated ruboxistaurin increased the intestinal I/R-induced downregulation of MnSOD, a primary ROS scavenging enzyme, but suppressed the accumulation of MDA, an indicator of lipid peroxidation (Figures 5a and b). Meanwhile, ruboxistaurin preserved intestinal I/R-induced GSH exhaustion and GSH-PX activity reduction (Figures 5c and d). Furthermore, the apoptosis execution enzyme caspase-3 has a crucial role in cell apoptosis by resulting in DNA fragmentation, degradation of cytoskeleton, and formation of apoptotic bodies. Arany *et al.*^30^ showed that p66^Shc^ was associated with cytochrome c, which is responsible for the activation of caspase-3 in the kidneys of mice with I/R injury. Our data showed that ruboxistaurin significantly attenuated intestinal caspase-3 activity and inhibited the apoptosis of the intestine subjected to I/R (Figures 6a and b). Therefore, it is conceivable that the inhibition of PKC*β*_2_ activation by ruboxistaurin attenuates p66^Shc^-mediated oxidative stress and subsequent apoptosis in intestinal I/R.

During the reperfusion period, mucosal barrier integrity is destroyed and the systemic release of pro-inflammatory cytokines occurs, with concurrent leukocyte activation and bacterial translocation.^31^ In this study, intestinal I/R injury significantly increased the serum levels of TNF-*α* and IL-6, suggesting that a severe systemic inflammation response was induced during the reperfusion period. Ruboxistaurin administration almost abrogated the increase in TNF-*α* and IL-6 serum concentration (Figure 2c).

Ruboxistaurin, an oral PKC*β* inhibitor, is currently undergoing phase 2 and phase 3 clinical testing for several cardiovascular diseases, such as diabetic retinopathy and diabetic kidney disease.^32, 33^ Due to be administrated orally, ruboxistaurin was gavaged for 3 days before I/R, which would be a potential limitation in acute clinical cases. However, the focus of this study was to investigate the role of PKC*β*_2_ in regulating p66^Shc^-mediated intestinal I/R injury.

In summary, our results demonstrate that the inhibition of PKC*β*_2_ activation attenuated intestinal I/R injury and systemic inflammation response by inhibiting the adaptor p66^Shc^-mediated oxidative stress and subsequent apoptosis. Furthermore, the activated principal isoform of PKC in intestinal I/R was specifically PKC*β*_2_, not PKC*β*_1_, PKC*δ*, or PKC*ɛ*. These may represent a novel therapeutic avenue for intestinal I/R injury.

## Materials and Methods

### Murine model of intestinal I/R

Male ICR mice (aged 4 weeks) weighing 20±2 g were obtained from the Animal Center of Dalian Medical University (Dalian, China), and kept under standard laboratory conditions with standard laboratory chow and water. The mouse intestinal occlusion-and-reperfusion procedure was performed as described previously.^5^ Briefly, the superior mesenteric artery was occluded by a microvascular clamp for 45 min and then 45, 90, or 180 min reperfusion was performed. Normal saline and ruboxistaurin (LY 333531; ENZO, Lausen, Switzerland) were given by oral gavage before sham and 45 min ischemia, followed by 90 min reperfusion at a dose of 10 mg/kg daily for 3 days (demonstrated to adequately inhibit PKC*β* activation in mice heart and vasculature).^9^ All procedures were conducted according to the Institutional Animal Care Guidelines, and were approved by the Institutional Ethics Committee.

### Histological and TUNEL staining

For histological and TUNEL analysis, formalin-fixed tissues were embedded in paraffin and sectioned. The 4-*μ*m sections were stained by hematoxylin–eosin. Intestinal I/R-induced mucosal injury was evaluated according to Chiu's score.^34^ TUNEL staining was performed using an apoptosis assay kit (Roche, Mannheim, Germany) according to the manufacturer's instructions.

### Measurement of cytokines

The levels of serum TNF-*α* and IL-6 were measured using Enzyme-linked immunosorbent assay (ELISA) kits (ENGTON Bio-engineering Limited Company, Shanghai, China), according to the manufacturer's protocols.

### Intestinal GSH, GSH-PX, and MDA activity assay

The GSH and GSH-PX activities were determined using an assay kit (Nanjing Jiancheng Corp., Nanjing, China), according to the manufacturer's recommendations. The level of MDA in the intestinal tissues was quantified by a lipid peroxidation MDA assay kit (Beyotime Institute of Biotechnology, Jiangsu, China) according to the manufacturer's protocol.

### Cell culture

Caco-2 cells were cultured at 37 °C in a humidified atmosphere of 5% CO_2_ in DMEM, supplemented with 10% fetal bovine serum, 1% non-essential amino acids, and 1% glutamide (Gibco, Carlsbad, CA, USA). To simulate physiologic conditions, Caco-2 cells were grown as monolayers on platforms, providing both apical and basolateral areas, thereby allowing cells to become polarized. The culture medium was then replaced with serum-free DMEM before experimental treatment.

### Transient transfection of siRNA

Caco-2 cells (1 × 10^5^) were seeded on six-well plates and transfected at the time of 70–80% confluence with a PKC*β*_2_ siRNA or non-binding control siRNA using Lipofectamin 2000 (Invitrogen, Karlsruhe, Germany), according to the manufacturer's instructions. The siRNA which was used to target PKC*β*_2_ had the sequences: 5′-GCGACCUCAUGUAUCACAUdTdT-3′ and 5′-AUGUGAUACAUGAGGUCGCdTdT-3′ (Genepharma, Shanghai, China). Scrambled siRNA which was used as a negative control had the sequences: 5′-ACGUGACACGUUCGGAGAAdTdT-3′ and 5′-UUCUCCGAACGUGUCACGUdTdT-3′. Commercial PKC*β*_2_ siRNA was utilized for the inhibition of PKC*β*_2_ expression as per manufacturer's protocol.

### H/R incubation and PMA exposure

To simulate *in vivo* intestinal ischemia, unless otherwise noted, cellular hypoxic conditions were created. For the hypoxic conditions, cells were incubated in a microaerophilic system (Thermo Fisher Scientific 8000, Marietta, GA, USA) at 5% CO_2_ and 1% O_2_, and balanced with 94% N_2_ gas for 15 h.^35^ The cells were then cultured in normoxic conditions for 6 h of reoxygenation. After transfection with control or PKC*β*_2_ siRNA, cells were incubated in either normoxic or H/R DMEM medium. Caco-2 cells were exposed to 100 nM PMA (Sigma-Aldrich, St. Louis, MO, USA) for 30 min in the absence or in the presence of PKC*β*_2_ siRNA or ruboxistaurin (20 nM).

### Western blot analysis

Equal protein amounts from isolated intestinal tissue and Caco-2 cell homogenate were removed using 10–15% SDS-PAGE (Bio-Rad, Hercules, CA, USA), and subsequently transferred onto PVDF membrane (Millipore, Bedford, MA, USA). Antibodies used for western blotting included those for PKC*β*_1_, PKC*β*_2_, and *β*-actin (Santa Cruz Biotechnology, Santa Cruz, CA, USA); PKC*δ*, PKC*ɛ*, cleaved caspase-3, and Na,K-ATPase (Bioworld Technology, St. Louis Park, MN, USA); phospho-PKC*β*_2_ (Cell Signaling Technology, Danvers, MA, USA); and p66^Shc^, phospho-p66^Shc^, and MnSOD (Abcam Ltd., Cambridge, UK). Appropriate secondary antibodies were used to detect the primary antibody/antigen complexes. The membranes were exposed to enhanced chemiluminescence-plus reagents (Beyotime Institute of Biotechnology). Emitted light was documented using a multispectral imaging system (UVP, Upland, CA, USA), and gels were analyzed using a Gel-Pro Analyzer, Version 4.0 (Media Cybernetics, Rockville, MD, USA).

### Statistical analysis

Densitometry was obtained by the image analysis software (UVP). All values are presented as means±S.E.M. The data were analyzed with a two-tailed Student's *t*-test when comparing means between two groups. One-way analysis of variance (ANOVA) followed by Student–Newman–Keuls (SNK) test was used when comparing multiple groups. The ordinal values of the gut injury scores were analyzed by the Kruskal–Wallis non-parametric test. Statistical analysis was performed by the GraphPad Prism (version 5.0; GraphPad Prism Software, La Jolla, CA, USA). *P*-values less than 0.05 were considered as significant.

## Abbreviations

I/R: ischemia/reperfusion
H/R: hypoxia/reoxygenation
TNF-*α*: tumor necrosis factor-*α*
IL-6: interleukin 6
siRNA: small interfering RNA
PMA: Phorbol 12-myristate 13-acetate
MnSOD: manganese superoxide dismutase
MDA: malondialdehyde
GSH: glutathione
GSH-PX: glutathione peroxidase
ROS: reactive oxygen species
